# Implicit Theories of Intelligence and Academic Achievement: A Meta-Analytic Review

**DOI:** 10.3389/fpsyg.2018.00829

**Published:** 2018-06-05

**Authors:** Ana Costa, Luísa Faria

**Affiliations:** Faculty of Psychology and Education Sciences, University of Porto, Porto, Portugal

**Keywords:** implicit theories of intelligence, incremental, entity, self-beliefs, growth mindset, academic achievement, students, meta-analysis

## Abstract

The current study intended to model the link between implicit theories of intelligence (ITI) and students' academic achievement, within a meta-analytic review procedure. To assess studies' effect size, the Pearson's correlation coefficient (*r*) was used. The review of 46 studies (94 effect sizes) with 412,022 students presented a low-to-moderate association between the ITI and students' academic achievement. The results indicated that incremental theorists are more likely to have higher grades in specific subjects (verbal and quantitative) and in overall achievement. The entity beliefs were positively associated with students' specific verbal and quantitative domains but at a lower magnitude than incremental beliefs. Moreover, the moderator effect analyses results indicated that the link between ITI and students' achievement was not moderated by gender, but there was a moderate association in student's middle school grade. Additionally, the ITI assessment based on the most recent versions of Dweck's scales, the use of specific academic scales instead of general ITI scales, and the use of the original measures rather than adapted versions strongly moderated the link between ITI and achievement. Moreover, students from Eastern continents (Asia and Oceania) reported a positive association between incremental beliefs and achievement, Europe displayed a positive link between entity beliefs and achievement, whereas North America presented negative correlations between entity perspectives and academic achievement. This meta-analysis updates the current evidence supporting the direct link of ITI and students' academic achievement and acknowledges specific effects that ITI could have in different academic outcomes.

## Introduction

Despite the fact that general intelligence predicts significant life outcomes (Neisser et al., 1996), recent literature has proven the crucial role of motivational patterns as sources of interindividual variability in different settings (Sorrentino and Higgins, 1986; Pintrich and Schunk, 1996; Efklides et al., 2001). The search for potential determinants of achievement that can be fostered at the individual or collective level raised particular interest in academics and researchers.

In the case of general intelligence, the individual's implicit beliefs about whether intelligence is malleable or fixed can have significant effects on academic and emotional outcomes (Aronson et al., 2002; Burnette et al., 2013; Romero et al., 2014). The fact that implicit theories of intelligence (ITI) tend to influence student's achievement particularly in challenging and demanding academic situations (Blackwell et al., 2007) constitutes an important protective academic factor.

Though ITI are hypothesized to correlate with student's academic achievement, the magnitude of this effect across the vast literature and the exploration of relevant moderators of this model still lacks systematization. Therefore, the present meta-analytic review intends to shed light on the influence of implicit theories of intelligence on students' academic achievement.

## Theoretical framework

While research on implicit theories have captured the interest of researchers in several domains (Aronson et al., 2002; Knee et al., 2003; Spinath et al., 2003; Tamir et al., 2007; Chen et al., 2008), continuous research on ITI conveys the important effect that this implicit factor can have on academic and emotional functioning (Cohen et al., 2006; Blackwell et al., 2007; Walton and Cohen, 2011; Yeager et al., 2013).

Individuals can typically perceive intelligence as more of a fixed unchanging characteristic (entity theory or beliefs) while others consider it as something that is malleable and prone to development (incremental theory or beliefs or growth mindset). Thus, individuals who hold entity or fixed theories of intelligence tend to believe that skills and abilities are relatively stable (Dweck, 1999) and that their performance is a consequence of that stability (Hong et al., 1999). Accordingly, they are likely to adopt performance goals focused at demonstrating their abilities and achieve positive evaluations from others (Dweck, 1999; Pepi et al., 2015). Individuals who hold an incremental or a growth mindset believe that these characteristics can change with effort and through time are more likely to adopt learning goals, choose challenging tasks, and employ adaptive strategies to improve their abilities (Dweck, 1999). The schematic knowledge structures that incorporate these beliefs (Ross, 1989) function as “implicit theories” since they are mostly unconscious to the individuals.

In the academic context, implicit theories about intelligence (see the achievement motivational model; Dweck and Leggett, 1988; Dweck, 1999) have been widely examined with respect to the learning processes and outcome variables (Burnette et al., 2013). Implicit theories can frame a student's specific mindset, along a continuum from an entity to an incremental belief, create distinct meaning systems (Hong et al., 1999) that can trigger different patterns of response to challenging situations and setbacks (Dweck and Leggett, 1988; Henderson and Dweck, 1990; Dweck, 1999; Dweck and Sorich, 1999) and ultimately influencing students' learning processes and achievement outcomes.

Although implicit theories of intelligence tend to be uncorrelated with general cognitive ability (Dweck et al., 1995; Robins and Pals, 2002), the literature has explored the effects that entity or incremental beliefs can have on students' academic outcomes (e.g., Stipek and Gralinski, 1996; Hong et al., 1999; Robins and Pals, 2002). On the one hand, entity theorists, who believe that intelligence is relatively fixed and predetermined, tend to adopt more performance goals (Dweck and Leggett, 1988) and prioritize positive assessment over learning (Elliott and Dweck, 1988; Robins and Pals, 2002). Additionally, they tend to attribute poor performance to lack of ability and therefore do not address poor performance with effort (Hong et al., 1999), assuming helpless strategies (Robins and Pals, 2002) and attributions of failure in face of setbacks (Henderson and Dweck, 1990), contributing to their academic helpless behavior. On the other hand, incremental theorists tend to focus more on learning goals (Dweck and Leggett, 1988), prioritize their intellectual development (Elliott and Dweck, 1988; Robins and Pals, 2002), value effort (Hong et al., 1999), and use mastery-oriented response patterns (Henderson and Dweck, 1990; Robins and Pals, 2002). Since incremental theorists believe that intelligence can be developed, they tend to increase effort in challenging situations to overcome difficulties, which will conduct them to develop their skills or the acquisition of new abilities. Incremental theorists attribute poor performances to lack of effort, rather than ability, and respond to poor performance with intentional remediation actions (Hong et al., 1999).

## Implicit theories of intelligence and academic achievement

Research has shown that there are positive effects of students' implicit theories of intelligence on their academic outcomes (Dweck, 2006; Burnette et al., 2013). Actually, research exploring the different response patterns of students' incremental and entity theories found that a more malleable or dynamic theory of intelligence tends to be associated with higher levels of academic engagement (Martin et al., 2013), learning goals in a growth oriented perspective (Dweck, 1999), mastery-oriented strategies (Burnette et al., 2013), overcoming domain-specific deficits (Alesi et al., 2016), academic achievement (Burnette et al., 2013) and fewer self-handicapping behaviors (Martin et al., 2001).

Dweck and Leggett (1988) suggested that implicit theories should indirectly predict achievement by influencing certain self-regulatory processes in response to ego threats (Dweck, 1999). Accordingly, implicit theories will theoretically support a weak direct association with academic achievement (Burnette et al., 2013). Nonetheless, research has provided evidence that implicit theories do directly predict achievement (e.g., Blackwell et al., 2007; Romero et al., 2014; Müllensiefen et al., 2015), although at a low magnitude (Burnette et al., 2013).

## Moderators of implicit theories of intelligence in the academic context

Research in the area of ITI has explored the impact that particular variables have on the association between ITI and students' academic performance. Considering gender differences, some studies have been associating the most entity beliefs of intelligence and ability with girls (Dweck, 1999; Pepi et al., 2006). Even when comparing high-achieving students (8th graders), girls were more likely to display a fixed or entity perspective of intelligence than an incremental one (Henderson and Dweck, 1990).

Additionally, some studies have shown that incremental and entity theorists might not present differences in their prediction of academic achievement, but this difference can surface when facing academically challenging situations (e.g., Blackwell et al., 2007). Research has highlighted that in particularly challenging academic cycles, such as junior high school, incremental theories of intelligence can present a leverage effect on a student's learning processes over time (Blackwell et al., 2007). Additionally, in college, incremental theories can predispose students to increased help-seeking behaviors, which will protect their academic achievement (Shively and Ryan, 2013). Moreover, implicit theories of intelligence can also have a crucial role in specific challenging subjects, such as math (Blackwell et al., 2007; Romero et al., 2014; Bostwick et al., 2017), because students are more prone to elicit effort and investment to overcome difficulties in the learning process. In general, in a stressful or demanding situation, students with a more malleable perspective of intelligence are more likely to adapt and succeed.

Because students' implicit theories of intelligence can vary across academic domains (Dweck, 1999) and because domain-specific beliefs tend to be greater predictors of goals, attributions and academic performance than unspecific or general ones (Bandura, 1997, 2006), current research has addressing the possible differential impact that specific academic subjects and performance-based (ability) ITI can have on academic performance and outcomes (e.g., Shively and Ryan, 2013; Chen and Tutwiler, 2017; Gunderson et al., 2017; Priess-Groben and Hyde, 2017). Additionally, since the conception of intelligence can diverge significantly across cultures (Furnham, 2000; Rammstedt and Rammsayer, 2000), implicit theories of intelligence, which refer to the way people perceive and evaluate both their own and others' intelligence, can be culturally shaped (Dweck and Leggett, 1988). Moreover, the research of Lim et al. (2002) found that African and Asian participants prioritized social aspects of intelligence and matters that facilitate interpersonal and group relations, whereas the Westerns, valued more references to the classic academic subjects (e.g., mathematics). These results highlighted possible conceptual intelligence differences across societies more collectivist or individualistic. Thus, exploring implicit theories of intelligence across cultures can help understand developmental and cultural differences in expectations about intellectual abilities (Sternberg, 2000). However, the effect of implicit theories on different academic subjects, considering relevant socio-demographic, academic, and cultural moderators still lacks systematization.

## Objectives

The current study intends to model the link between ITI and students' academic achievement using a meta-analysis procedure. In particular, studies were reviewed to (a) provide estimates of the effect size of correlations between incremental and entity theories of intelligence and students' academic achievements (verbal, quantitative, general assessment, and self-reported grades) and (b) analyze whether the links between implicit theories of intelligence and students' achievement in different subjects are moderated by students' gender, educational level, ITI measure used, type of implicit theories measure (general or specific; original or adapted version), and student's cultural background.

## Methods

This study followed recommended guidelines stated in the Preferred Reporting Items for Systematic Reviews and Meta-Analyses (PRISMA; Moher et al., 2009).

### Eligibility criteria

To develop an extensive evidence base that could present the association between ITI and students' academic achievement, we did not limit our search strategy by language, publication status, country, or date. Studies were eligible for this review if they reported quantitative measures of academic achievement outcomes for students with implicit (incremental or entity) theories of intelligence. The eligible participants of this study were currently students attending any educational level (middle school, high school, and college). Quantitative measures of student academic achievement outcomes could include any form of achievement grade (e.g., language, literacy, reading, math, biology or GPA).

### Information sources and search strategy

To identify all eligible studies, a comprehensive literature search of the Educational Resource Information Center (ERIC), Fonte Acadêmica, PsycINFO, Academic Search Complete, Education Source and Psychology and Behavioral Sciences Collection databases was completed. The first author searched these databases using the key terms “implicit theories of intelligence” or “growth theories” or “growth mindset” or “incremental theories” or “self-theories of intelligence” or “personal conceptions of intelligence” and “academic achievement” or “academic success” or “academic performance” or “grades” or “GPA” (Grade Point Average).

This search included studies published before November 2017 with no limitations of language, country, or publication status. In addition to these electronic searches, the authors examined the websites and curricula vitae of the first authors of eligible studies (when available) and reviewed the references of all eligible studies by “back-tracking” in order to find potentially suitable articles that might have been overlooked during the initial search.

### Coding of variables

The following study characteristics were coded: identification data (author, year of publication), country where the study was developed, sample size, mean age of the participants, gender distribution (the percentage of male participants), participants' educational level, instrument used to assess ITI, type of measurement of ITI (incremental or entity), outcomes of academic achievement (verbal, quantitative, general assessment, and self-report achievement), and data needed for computing an effect size (all the included studies reported the correlational coefficient between ITI and academic achievement).

For the assessment of the implicit theories of intelligence, all the information was obtained through self-reporting measures, mainly derived from Dweck's (1999) Theories of Intelligence Scale (TIS), thus representing the same construct. This instrument assesses general beliefs about the fixedness or malleability of intelligence and consists of two subscales, measuring entity and incremental theories, with 4 items each. Research has indicated that TIS has good reliability (α from 0.82 to 0.97) and construct validity (Dweck et al., 1995). In some of the studies review, both incremental and entity subscales were included separately, while in others, one of the subscales was reverse coded, thus yielding a single measure for incremental theory. This fact allows exploration of the effects of the different subscales. Some studies used specific measurement of implicit theories based on Dweck's TIS applied to particular subjects, such as math. These studies were coded as specific-subject ITI.

### Outcome measures

The outcome academic achievement considered students' academic grades in different subjects. The studies in the final sample reported different academic achievement outcomes. When studies reported more than one outcome of interest, all relevant data from each study sample were extracted. Then, the authors classified outcomes into broad constructs and, subsequently, conducted separate meta-analyses for each of these constructs. All of the dependent variables reported the correlation coefficient with ITI, incremental and/or entity.

### Analytical strategies/procedure

The Pearson's correlation coefficient (*r*) was the measure of effect size used, given that all of the studies included in the meta-analysis directly reported this statistic to assess the outcomes of academic achievement. The Pearson's correlation coefficient can perform as effect size estimate (Borenstein, 2009) and considering Cohen's (1988) guidelines for interpreting effect sizes from *r*, a value below 0.1 indicates low, a value of 0.3 medium, while values above 0.5 indicate large effect sizes.

Considering that the studies in the meta-analysis come from different populations, we used random-effects models and the methods suggested by Hedges et al. (Cooper et al., 2009). The test of possible moderator's effects was decided based on the homogeneity test, which explored variance in effect sizes between different samples' characteristics. To assess study heterogeneity, *Q* statistics were examined, evaluating their statistical significance at a value of 0.05. When the homogeneity test was significant (*Q*_*BET*_ < 0.05), *post-hoc* moderator analyses were implemented to test whether the groups were significantly different.

We conducted *post-hoc* moderator analyses, examining the potential effects that reported gender (i.e., percent male of sample), educational level of sample (i.e., middle school, high school, college), ITI measure, type of ITI measure (i.e., general or specific ITI), version of instrument (adapted or original), and cultural background (e.g., Asia, Europe, North America, Oceania) have on the correlational coefficients. To assess publication bias and the possibility of small-study bias, we visually inspected funnel plots and conducted both an Egger test and Begg and Mazumdar's (1994) rank correlation test (Field and Gillett, 2010). Analyses were conducted using the Comprehensive Meta-Analysis Software (version 3).

## Results

### Study selection

**Figure 1:**
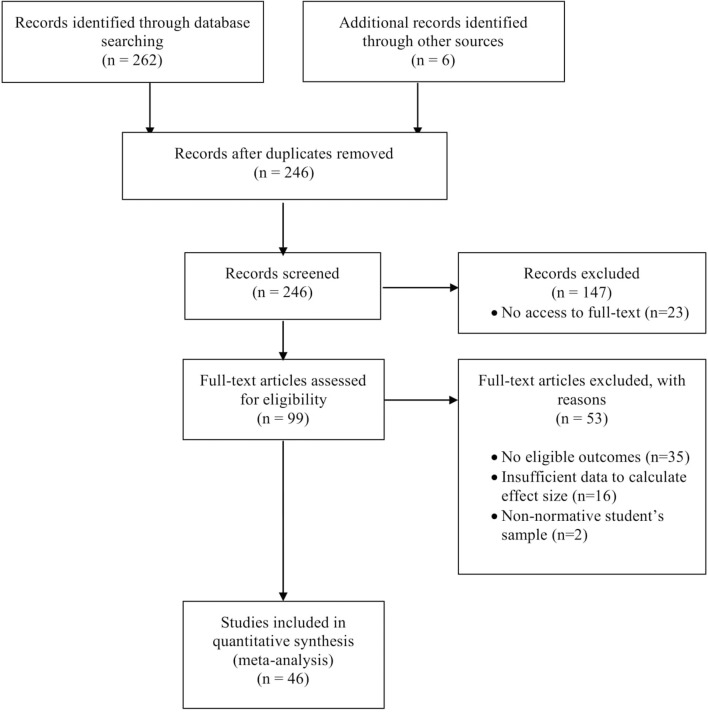
Flow diagram for studies included in the meta-analysis.

The electronic database search revealed 262 reports, and 6 additional reports were identified by back-tracking, of which 240 were unique citations reviewed at the title-abstract screening level. Twenty-three studies were not available in full-text and/or were not provided by the corresponding author. Following the title-abstract screening and inclusion of gray literature, we reviewed 99 reports at the full-text level. Our final sample of eligible studies consisted of 46 reports (cf. Figure 1) and 94 eligible outcomes.

Of the excluded studies, 35 reports provided no eligible academic achievement outcomes. For instance, some studies presented cognitive measures of achievement. Sixteen reports did not provide sufficient data to calculate the effect size. Two reports presented results for specific student's populations (e.g., gifted students) and were not included as normative student's populations, which was the eligibility criteria for participants in this study.

### Study characteristics

Characteristics of included studies are summarized in Table 1. The final sample consisted of 46 studies (*N* = 412,022) reported in journal articles (*k* = 39), dissertations (*k* = 6), and proceedings published in paper (*k* = 1), between 2002 and 2017.

**Table 1:** Studies included in the meta-analysis.

**Author (Year)**	**Sample (N)**	**Mean age (*SD*)**	**TOI measure**	**TOI factors**	**General or specific-domain measure**	**Academic achievement outcome**	**% male**	**Publication's type**
Aditomo, 2015	123 (high school)	18.67 (0.74)	Dweck et al., 1995	Incremental	General specific	Final exam score	19	Article
Blackwell et al., 2007	373 (middle school)		Dweck, 1999	Incremental	General	Maths achievement	47	Article
Bostwick et al., 2017	4,411 (middle school)	13.5 (0.95)	Dweck, 1999	Incremental	Specific	Maths achievement	54	Article
Burnette et al., 2017	183 (high school)	15.2	Dweck, 1999	Incremental	General	GPA	0	Article
Chen and Tutwiler, 2017	506 (middle);354 (high school)		Dweck, 1999	Incremental entity	Specific	GPA	59; 51	Article
Chen and Wong, 2014	312 (college)	19.88 (1.3)	Dweck, 1999	Incremental	General	GPA	40	Article
Chen and Wong, 2015	418 (college)	19.88 (1.3)	Dweck, 1999	Incremental	General	GPA	46	Article
Claro et al., 2016	168,203 (maths sample) 168,502 (language sample) (high school)		Dweck, 1999	Incremental	General	Maths achievement language achievement	49	Proceedings
Clevenger, 2014	208 (middle school)	11.86	Dweck, 1999	Incremental	General	GPA	43	Dissertation
Dai and Cromley, 2014	330 (college)	19.8 (1.6)	Dweck, 1999	Incremental entity	Specific	Biology course grade	42	Article
De Castella and Byrne, 2015	643 (high school)	16.6 (1.01)	Dweck, 1999	Incremental	General	Self-report grade	38	Article
Dickhäuser et al., 2016	288 (high school)	17.7	Spinath and Schöne, 2003	Incremental	General	GPA	43	Article
Dinger et al., 2013	524 (high school)	17.43 (1.12)	Spinath and Schöne, 2003	Incremental	General	GPA	47	Article
Diseth et al., 2014	1,101 (middle school)		Dweck, 1999	Incremental	General	Self-report grade	49	Article
Dixson et al., 2017	105 (high school)	16.15 (1.08)	Dweck, 1999	Incremental	General	GPA	41	Article
Dupeyrat and Mariné, 2005	73 (high school)	31	Hong et al., 1995	Incremental entity	General	GPA	42	Article
Fabert, 2015	489 (college)		Hong et al., 1995	Incremental	General	GPA	64	Dissertation
Gonida et al., 2006	232 (middle school)	11.4	Henderson et al., 1992	Incremental	General	GPA	50	Article
Gunderson et al., 2017	82 (middle); 140 (high school); 190 (college)		Stipek and Gralinski, 1996; Dweck, 1999; Furnham et al., 2002	Incremental	General specific	Maths achievement reading-writing achievement	35; 39; 56	Article
Jones et al., 2012	163 (middle school)		Dweck, 1999	Incremental	Specific	Maths achievement	45	Article
King, 2012	676 (high school)	14.84 (1.01)	Dweck, 1999	Incremental entity	General	GPA	42	Article
Kornilova et al., 2009	300 (college)	19.48 (1.98)	Dweck, 1999	Incremental	General	Final exam score GPA	26	Article
Leondari and Gialamas, 2002	451 (middle school)	12.15	Stipek and Gralinski, 1996	Incremental	General	GPA (math and language grade average)	45	Article
Li et al., 2017	4036 (high school)	15.41 (0.45)	Hong et al., 1999	Incremental	General	GPA (language maths and English grade average)	51	Article
Luo et al., 2014	273 (middle school)	14.39 (0.44)	Dweck, 1999	Incremental	Specific	Maths achievement	36	Article
Magno, 2012	291 (college)	19.09 (1.25)	Abd-El-Fattah and Yates, 2005	Incremental entity	General	GPA	74	Article
Miller, 2011	123 (college)	18.71	Hong et al., 1995	Incremental entity	General	Final course GPA	57	Dissertation
Müllensiefen et al., 2015	312 (middle and high school)	14.14 (1.92)	Dweck, 1999	Incremental	General	GPA	0	Article
Northrop, 2015	26 (high school)		Spinath et al., 2003	Incremental	General	Self-report GPA	23	Dissertation
Ollfors and Andersson, 2007	918 (high school)		Dweck, 1999	Incremental	General	GPA	46	Article
Pepi et al., 2006	730 (Portuguese); 814 (Italian sample) (high school)	19.07; 18.26	Faria, 2006	Incremental entity	General	Language achievement maths achievement GPA	39; 43	Article
Phillips-Martinez, 2018	55 (middle and high school)		Dweck, 2006	Incremental	General	Final course GPA	17	Dissertation
Priess-Groben and Hyde, 2017	165 (high school)		Chiu et al., 1997	Incremental	Specific	Maths achievement	53	Article
Renaud-Dubé et al., 2015	650 (high school)	14.86 (1.36)	Henderson et al., 1992	Incremental	General	Self-report GPA	54	Article
Rickert et al., 2014	142 (high school)	14.95 (0.40)	Dweck, 1999	Incremental	General	GPA	42	Article
Robins and Pals, 2002	363 (high school and college)		Erdley and Dweck, 1993	Incremental	General	GPA high school GPA College		Article
Romero et al., 2014	115 (middle school)		Dweck, 1999	Incremental	General	GPA Maths advanced course grade	42	Article
Shih, 2007	298 (middle school)	11.5	Dweck, 1999	Incremental entity	General	GPA	52	Article
Shively and Ryan, 2013	159 (college)		Dweck, 1999	Incremental	General specific	Maths course grade	40	Article
Stocker et al., 2010	385 (high school)	16.3 (1.25)	Faria, 2006	Incremental entity	General	Language achievement Maths achievement GPA	44	Article
Tarbetsky et al., 2016	174 (middle school)	13.67 (1.00)	Dweck, 1999	Incremental	General specific	Literacy achievement Maths achievement GPA	63	Article
Tempelaar et al., 2015	4,594 (college)	20.21	Dweck, 1999	Incremental entity	General	Maths exam grade Statistic exam grade Social science exam grade	63	Article
Volpe, 2017	307 (middle school)		Dweck, 1999	Incremental	General	Language achievement Maths achievement GPA	46	Dissertation
West et al., 2016	1,340 (middle school)		Dweck, 2006	Incremental	General	English, Language, Arts achievement Maths achievement		Article
Yeager et al., 2016	6,883 (middle school)		Dweck, 1999	Incremental	General	Self-report GPA	52	Article

Included studies ranged widely in sample size (from *n* = 26 to *n* = 168,552), but both the mean sample size (*N* = 4,572) and the median sample size (*N* = 730) demonstrate that most studies (*k* = 34) drew from samples of over 500 students. On average, the study samples were approximately 45.5% male-identified (*k* = 44), with a mean age of 16.25 (*k* = 28) and included different educational level samples (middle school *n* = 18, high school *n* = 22 and college *n* = 11). One study drew from a national sample (Claro et al., 2016). All studies used surveys to collect students' self-reports of ITI. Most studies were written in English and were conducted in several countries (e.g., Chile, Portugal, Indonesia, Australia, China, EUA, Germany).

### Synthesis of results

After completing data extraction, students' academic achievement was classified into the following four constructs: verbal (*k* = 14), quantitative (*k* = 26), general assessment (*k* = 45), and self-reported achievement (*k* = 5). We completed a principal meta-analysis for the effect of ITI on students' academic achievement and then four meta-analyses for each outcome construct.

Finally, we conducted *post-hoc* moderator analyses to examine the effects of the following on the findings: reported gender (i.e., percent male of sample), educational level of sample (i.e., middle school, high school, college), ITI measure used, type of ITI measure (i.e., general or specific-subject), version of ITI instrument (original version or a translated and adapted version of the original instrument), and cultural background (e.g., North America, Europe, Asia) as well as the Egger test for small-study bias and Begg and Mazumdar's (1994) rank correlation test to confirm publication bias.

### Academic achievement

The mean weighted effect size of 46 studies (*N* = 412,022; 94 effect sizes) was significantly positive, *r* = 0.07, 95% confidence interval (CI) [0.04, 0.11], *z* = 4.19, *p* < 0.001, *Q*_W_ (93) = 6900.52, *pQ*_*W*_ < 0.001, indicating that ITI are, in general, positively related to academic achievement at a low magnitude. Specifically, the aggregated studies data in achievement domains (cf. Table 1) provided positive and significant associations of ITI and students' verbal (*r* = 0.13; 95% CI = 0.04, 0.22; *p* < 0.001), quantitative (*r* = 0.12; 95% CI = 0.06, 0.18; *p* < 0.001) and general grade assessment (*r* = 0.06; 95% CI = 0.01, 0.10; *p* < 0.001), but showed no significant relation with self-reported grades (*r* = −0.10; 95% CI = −0.03, 0.05; *p* = 0.190).

Additionally, incremental and entity perspectives of intelligence were examined across studies to explore the possible differential association with students' academic achievement. While the incremental beliefs were positively related with students' achievement (*k* = 74; *r* = 0.10; 95% CI = 0.07, 0.14; *p* < 0.001), the entity perspective was not (*k* = 20; *r* = −0.03; 95% CI = −0.08, 0.03; *p* = 0.315). Moreover, since the two factors of ITI (incremental and entity) provided significant differences in their association with academic achievement *Q*_*BET*_(*1*) = 15.40, *p* < 0.001 and to prevent masking possible differential effects of the two types of beliefs, the authors decided to proceed with independent analyses of the two perspectives and the academic achievement outcomes.

#### Verbal domain

**Figure 2:**
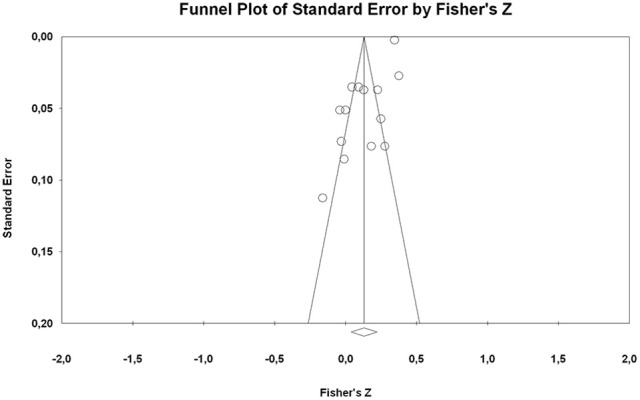
Funnel plots of effect sizes of the correlation between ITI and student's verbal domain achievement.

Seven studies in the sample reported a language learning related outcome: 6 language, 1 literacy, 3 writing-reading achievement. The aggregated data across studies indicated that the incremental beliefs are positively related to higher levels of verbal achievement (*k* = 11, *r* = 0.15, 95% CI = 0.05, 0.24; *p* = 0.004). The entity perspective was also associated with verbal academic outcomes but at a lower level (*k* = 3, *r* = 0.08, 95% CI = 0.02, 0.15; *p* = 0.016). Regarding publication bias, the inspection of the funnel plot demonstrated that the studies dispersion was not completely symmetrical (cf. Figure 2). Additionally, the results of the Egger test for small-study bias was significant (*p* < 0.001), meaning that there was evidence that the effect sizes provided in the verbal domain were impacted by the omission of small studies. However, Begg and Mazumdar's rank correlation test displayed no significant differences (*p* = 0.912), counter to the indication of possible bias.

#### Quantitative domain

A total of 14 studies provided data related to the effect of ITI in students' quantitative-related domains (12 math achievement, 1 advanced math course grade, 1 statistics exam grade, 1 math exam grade). Synthesizing findings across studies, the incremental and entity beliefs were associated with increased levels of quantitative domain achievement (*k* = 21, *r* = 0.14, 95% CI = 0.06, 0.21; *p* = 0.001 and *k* = 5, *r* = 0.07, 95% CI = 0.04, 0.10; *p* < 0.001, respectively).

**Figure 3:**
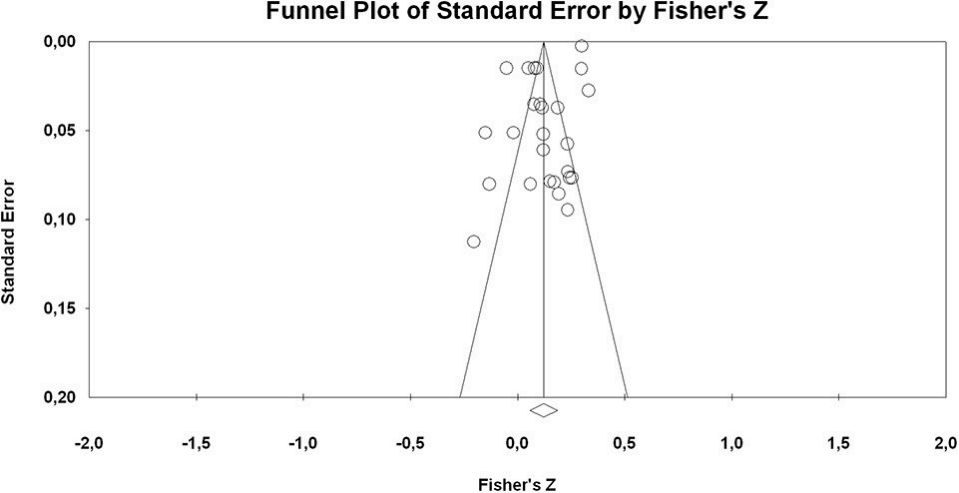
Funnel plots of effect sizes of the correlation between ITI and student's quantitative domain achievement.

The funnel plot was not completely symmetrical (cf. Figure 3), and the results of the Egger test were significant (*p* = 0.002), which indicated possible bias. However, the Begg and Mazumdar's rank correlation test were non-significant (*p* = 0.108).

#### General assessment

**Figure 4:**
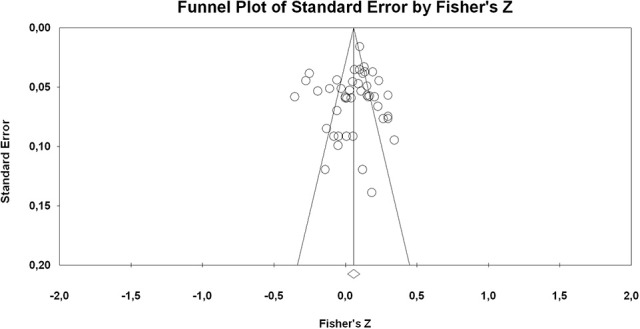
Funnel plots of effect sizes of the correlation between ITI and student's general assessment achievement.

Concerning students' general assessment outcomes, 29 studies in the sample provided data for the analysis (28 GPA, 2 final exam score, 2 final course grades, 2 mean of different subject's grades). The data reported indicated that the ITI are positively related to the students' general grade assessment (*k* = 35, *r* = 0.10, 95% CI = 0.07, 0.14; *p* < 0.001) and were significantly different from students' entity beliefs, which were not associated with the general grade assessment [*k* = 10, *r* = −0.10, 95% CI = -0.21, 0.02; *p* = 0.118; *Q*_*BET*(1)_ = 9.55, *p* = 0.002]. Concerning possible publication bias in the results of the general assessment grade, the inspection of the funnel plot (cf. Figure 4) and both non-significant Egger and Begg and Mazumdar's rank correlation test (*p* = 0.564, *p* = 0.611, respectively) showed the absence of possible bias.

#### Self-reported achievement

**Figure 5:**
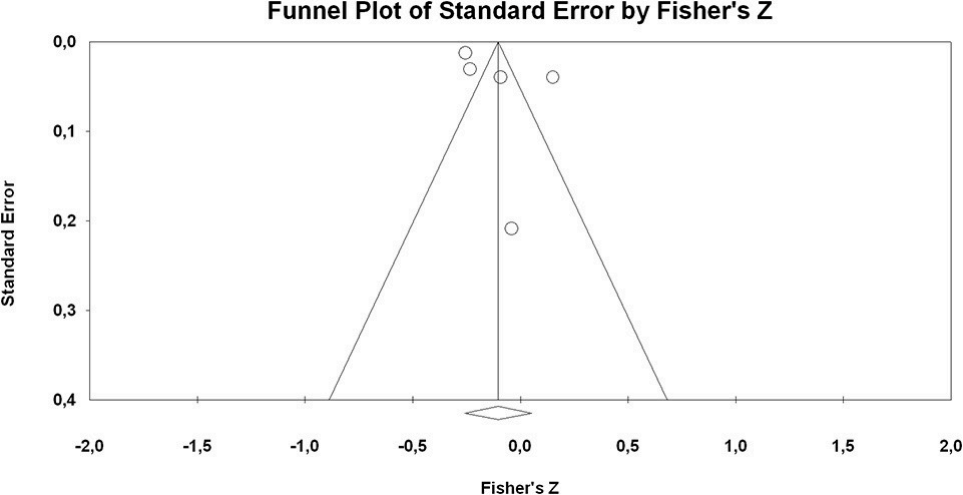
Funnel plots of effect sizes of the correlation between ITI and student's self-report achievement.

Five studies reported data to explore the association between ITI and students' self-reported grades (2 self-report grades, 3 self-report GPA). The available data were related only to incremental beliefs, which were not associated with students' self-reported achievement (*k* = 5, *r* = −0.10, 95% CI = −0.25, 0.05; *p* = 0.190). For publication bias analysis, the inspection of the funnel plot (cf. Figure 5) and the non-significant Egger test for small-studies bias (*p* = 0.253) and Begg and Mazumdar's test (*p* = 0.462) indicated evidence of no publication bias.

### Moderators analyses

Total homogeneity tests across incremental and entity ITI independent samples were performed. The results showed a significant homogeneity coefficient between ITI and student's academic achievement [*Q*_*T*(74)Inc_ = 5308.416, *p* < 0.001; *Q*_*T*(20)Ent_ = 267.557, *p* < 0.001]. These results indicate that students' gender, educational level, specificities of ITI measurement, students cultural background might moderate the links between ITI and student's academic outcomes. Therefore, meta-regression analyses to examine gender influence and meta-analysis of variance to examine whether educational level, ITI measurement or cultural background influenced the relation between ITI and achievement were conducted.

### Moderators analyses related to individual differences

#### Gender

A meta-regression analysis was computed in order to test the predictive value of gender upon the effect sizes on the data from the 44 studies that reported the proportion of gender. The meta-regression analysis (*Q*_*T*_ [1, *k* = 91] = 0.30, *p* = 0.581) showed that gender did not moderate the link between ITI and academic achievement, and thus, no subsequent moderator analyses on gender were performed.

#### Educational level

The results of the homogeneity test (*Q*_*BET*_ = 3402.636, *df* = 4, *p* < 0.001) suggested that the link between ITI and academic achievement was influenced by educational levels. Actually, students' incremental beliefs are associated with higher general achievement in different educational levels (middle school *k* = 26, *r* = 0.15, 95% CI = 0.04, 0.26; *p* = 0.008; high school *k* = 30, *r* = 0.09, 95% CI = 0.05, 0.12; *p* < 0.001; college *k* = 16, *r* = 0.06, 95% CI = 0.01, 0.11; *p* = 0.031). Although there is a low association between incremental ITI and achievement, this relationship is stronger in the middle school. Entity theories were not associated with student's achievement in college (*k* = 6, *r* = −0.01, 95% CI = −0.07, 0.06; *p* = 0.875) and high school (*k* = 12, *r* = 0.01, 95% CI = −0.07, 0.09; *p* = 0.776). Due to insufficient studies the effect size for middle school was not computed (*k* = 2).

Concerning specific domains of achievement, incremental beliefs were related to higher verbal achievement in the middle school (*k* = 5, *r* = 0.21, 95% CI = 0.07, 0.35; *p* = 0.004) but not in high school (*k* = 5, *r* = 0.12, 95% CI = −0.06, 0.29; *p* = 0.180), with higher levels of quantitative academic outcomes in the middle school (*k* = 10, *r* = 0.20, 95% CI = 0.14, 0.27; *p* < 0.001) but not in others educational levels (high school *k* = 6, *r* = 0.13, 95% CI = −0.02, 0.26; *p* = 0.081; college *k* = 5, *r* = 0.04, 95% CI = −0.06, 0.14; *p* = 0.448), and with student's general assessment in middle school (*k* = 9, *r* = 0.19, 95% CI = 0.12, 0.26; *p* < 0.001), high school (*k* = 16, *r* = 0.06, 95% CI = −0.07, 0.06; *p* = 0.016), and college (*k* = 8, *r* = 0.09, 95% CI = 0.01, 0.16; *p* = 0.026) but not with self-reported grades (*k* = 3, high school *r* = 0.06, 95% CI = 0.01, 0.11; *p* = 0.016). Studies that explored entity beliefs used only high school educational level samples, and they were associated with verbal achievement (*k* = 3, *r* = 0.08, 95% CI = 0.02, 0.15; *p* = 0.016) and quantitative achievement (*k* = 3, *r* = 0.08, 95% CI = 0.00, 0.15; *p* = 0.048) but not associated with students' general assessment (*k* = 6, *r* = −0.06, 95% CI = −0.20, 0.09; *p* = 0.437).

### Moderators analyses related to ITI measurement

#### General vs. specific ITI

Ten studies used specific ITI measures to assess students' beliefs in particular academic subjects. The results of the homogeneity test (*Q*_*BET*_ = 110.058, *df* = 1, *p* < 0.001) suggested that the link between ITI and academic achievement was influenced by the type of ITI measurement. The results indicated that both incremental and entity beliefs, when measured by specific ITI scales, have a greater association (from low to moderate) with student's academic achievement (*k* = 18, *r* = 0.13, 95% CI = 0.07, 0.19; *p* < 0.001; *k* = 3, *r* = −0.27, 95% CI = −0.35,−0.18; *p* < 0.001, respectively), than when assessed by the general ITI scales (*k* = 56, *r* = 0.10, 95% CI = 0.05, 0.14; *p* < 0.001; *k* = 17, *r* = 0.02, 95% CI = −0.03, 0.07; *p* = 0.404, respectively).

Due to the limited number of studies that provided effect sizes for the association of specific entity beliefs and specific domains of achievement, analyses were conducted only for the incremental theories. The findings indicated that specific incremental theories are more associated with students' quantitative (*k* = 9, *r* = 0.16, 95% CI = 0.07, 0.24; *p* = 0.001) and global assessment grades (*k* = 4, *r* = 0.17, 95% CI = 0.08, 0.26; *p* < 0.001) than are the general incremental theories *(k* = 12, *r* = 0.12, 95% CI = 0.02, 0.23; *p* = 0.024; *k* = 31, *r* = 0.09, 95% CI = 0.06, 0.13; *p* < 0.001, respectively). Students' verbal achievement was associated with the incremental general ITI measurement (*k* = 7, *r* = 0.21, 95% CI = 0.11, 0.31; *p* < 0.001) but not with the specific ITI measurement (*k* = 4, *r* = 0.01, 95% CI = -0.13, 0.14; *p* = 0.915).

#### ITI measures

Since the homogeneity test indicated sources of variability explained by the different ITI measurement scales (*Q*_*BET*_ = 5360.410, *df* = 14, *p* < 0.001), this moderator effect was assessed. However, due to the reduced number of studies for each scale, the analysis conducted was limited to the effect of the type of measurement used upon the association of both incremental and entity theories and students' achievement. The results indicated that the association between ITI (incremental and entity) and students' academic achievement was moderated by the Dweck's TIS scale (version 1999, *k* = 54, *r* = 0.13, 95% CI = 0.11, 0.16; *p* < 0.001 and version 2006, *k* = 3, *r* = 0.35, 95% CI = 0.30, 0.40; *p* < 0.001, respectively), and Personal Conceptions of Intelligence (Faria, 2006; *k* = 18, *r* = 0.07, 95% CI = 0.03, 0.11; *p* = 0.002) but not by the Dweck et al. (1995; *k* = 5, *r* = 0.02, 95% CI = −0.05, 0.09; *p* = 0.544) scale.

#### Original vs. adapted ITI measure

The results of the homogeneity test (*Q*_*BET*_ = 1348.737, *df* = 1, *p* < 0.001) suggested the influence that the original or the translated and adapted version of the instrument has on the effect sizes of the association of ITI and achievement. The results indicated that when using the original version of an ITI's instrument the association's magnitude of the incremental (*k* = 44, *r* = 0.11, 95% CI = 0.04, 0.19; *p* = 0.005) and entity theories (*k* = 4, *r* = −0.22, 95% CI = −0.33,−0.10; *p* < 0.001) with academic achievement is stronger, compared with the versions resulting of a process of translation or adaptation to a specific language and culture (incremental *k* = 30, *r* = 0.09, 95% CI = 0.04, 0.13; *p* < 0.001; entity *k* = 16, *r* = 0.02, 95% CI = −0.03, 0.07; *p* = 0.407).

In particular, incremental theories of intelligence assessed by either the original or the adapted version of the instrument were not related to students' verbal achievement (*k* = 7, *r* = 0.14, 95% CI = −0.01, 0.28, *p* = 0.066; *k* = 4, *r* = 0.15, 95% CI = −0.04, 0.32; *p* = 0.125, respectively), but for the quantitative achievement, the original version of ITI instrument proved to be associated with greater academic outcomes (*k* = 14, *r* = 0.17, 95% CI = 0.11, 0.24; *p* < 0.001; adapted version *k* = 7, *r* = 0.08, 95% CI = −0.07, 0.23; *p* = 0.272). Considering students' global assessment grade, the incremental (*k* = 18, *r* = 0.10, 95% CI = 0.04, 0.16; *p* = 0.002) and entity (*k* = 3, *r* = −0.17, 95% CI = -0.30, −0.04; *p* = 0.012) original version of ITI measurement and the incremental adapted version were related to achievement (*k* = 17, *r* = 0.11, 95% CI = 0.06, 0.15; *p* < 0.001; entity adapted version *k* = 7, *r* = −0.07, 95% CI = −0.21,.08; *p* = 0.366).

### Moderators analyses related to cultural background

The results of the homogeneity test (*Q*_*BET*_ = 5095.394, *df* = 5, *p* < 0.001) suggested that the link between ITI and achievement was influenced by the students' cultural background. The aggregated data across studies indicated that the incremental beliefs were associated with higher levels of students' achievement in Asia (*k* = 9, *r* = 0.12, 95% CI = 0.07, 0.17; *p* < 0.001), Oceania (Australia) (*k* = 8, *r* = 0.21, 95% CI = 0.09, 0.34; *p* < 0.001), and at the limit of significance in North America (*k* = 33, *r* = 0.10, 95% CI = −0.00, 0.19; *p* = 0.051) but were not significant for Europe (*k* = 20, *r* = 0.04; 95% CI = −0.01, 0.10; *p* = 0.119). However, entity theories of intelligence were negatively associated with student achievement in North America (*k* = 4, *r* = −0.22; 95% CI = −0.35, −0.10; *p* < 0.001) and positively associated with student achievement in Europe (*k* = 13, *r* = 0.07; 95% CI = 0.05, 0.10; *p* < 0.001) but were not significantly associated with achievement in Asia (*k* = 3, *r* = −0.19; 95% CI = −0.40, 0.02; *p* = 0.071).

Due to limit data of entity studies in different countries, some of the analyses for specific achievement domains were not computed. Considering the North American cultural background, the results highlighted the significant effect that incremental theories have on students' quantitative achievement (*k* = 11, *r* = 0.14; 95% CI = 0.04, 0.23; *p* = 0.004), but not for verbal (*k* = 5, *r* = 0.10; 95% CI = −0.11, 0.30; *p* = 0.348), general assessment (*k* = 13, *r* = 0.09; 95% CI = 0.02, 0.16; *p* = 0.014) and self-report (*k* = 3, *r* = −0.05; 95% CI = −0.39, 0.29; *p* = 0.771) outcomes. Europe presented a significant relationship between the incremental perspectives and the global assessment grade (*k* = 10, *r* = 0.08; 95% CI = 0.01, 0.15; *p* = 0.020) but not for more specific domains [verbal (*k* = 3, *r* = 0.08; 95% CI = −0.07, 0.23; *p* = 0.299) or quantitative (*k* = 5, *r* = 0.03; 95% CI = −0.06, 0.13; *p* = 0.483)], whereas for the entity perspective of intelligence, it was significant related to specific verbal (*k* = 3, *r* = 0.08; 95% CI = 0.02, 0.15; *p* = 0.016) and quantitative (*k* = 5, *r* = 0.07; 95% CI = 0.04, 0.10; *p* < 0.001) academic outcomes but not for the global assessment grade (*k* = 4, *r* = 0.05, 95% CI = −0.04, 0.14; *p* = 0.288). In the Asian cultural background, incremental perspective of intelligence was positively associated with the global assessment grade (*k* = 8, *r* = 0.12; 95% CI = 0.06, 0.18; *p* < 0.001), but no relationship was found with the entity beliefs (*k* = 3, *r* = −0.19; 95% CI = −0.40, 0.02; *p* = 0.071).

## Discussion

The present meta-analysis integrated 46 studies, which provided 94 independent effect sizes within a total sample of 412,022 students. The effect size of the correlations between ITI (incremental and entity beliefs) and students' academic achievement ranged from low to moderate. Additionally, this link was moderated by students' academic grade, ITI measurement (measure used; general or specific-subject ITI scale, original or adapted ITI scale version), and cultural background.

### ITI and student achievement

In this meta-analysis, in general, a significant yet low association between ITI and students' academic achievement was found. As expected, these results confirm most of the literature that observe that there is a positive direct association between students' ITI and their academic performance, although overall at a modest level (Blackwell et al., 2007; Burnette et al., 2013). In this study, in general, the ITI were not associated with students' self-report achievement.

Moreover, the results indicated that the link between ITI and achievement were positively significant for specific subjects, such as verbal and quantitative academic domains, and for general assessment grade, although the last at a lower magnitude. Research has highlighted that ITI can have particular importance in challenging academic situations. The fact that verbal and quantitative subjects constituted central and transversal domains across educational levels, eliciting from students' greater effort and time, might justify the increased association between ITI and those specific academic domains. In contrast, the global assessment grade combines several other academic subjects with possibly different links to students' ITI that could have narrowed that association.

Since incremental or entity beliefs of intelligence lead to different academic response patterns, and the association established with academic outcomes was significantly different in our study, those perspectives were independently explored. On one hand, the results indicated that incremental theorists are more likely to have higher grades in specific subjects (verbal and quantitative) and in overall achievement (*r* = 0.10 to 0.15, *p* < 0.001). On the other hand, the entity beliefs were positively associated with students' specific subjects' achievement, such as verbal and quantitative domains *(r* = 0.07 to 0.08, *p* < 0.001), although at a decreased magnitude compared to the dynamics beliefs, but were not associated with overall academic achievement. These results are consistent with the literature, which confirms that a students' incremental perspectives of intelligence could have a positive significant influence on academic achievement (Blackwell et al., 2007; Burnette et al., 2013; Romero et al., 2014; Bostwick et al., 2017). Although it was theoretically expected that the entity theories would present a negative association with achievement, some studies have reported similar positive results (Stocker et al., 2010), indicating that a more fixed perspective of intelligence, focused on the performance goals and outcomes, could currently foster adaptation and academic success. Perhaps more competitive educational systems might cultivate the entity perspectives of students, which perceived achievement (outcome) as the most important factor to proceed both academically and professionally.

### Moderator effects

In the current study, the moderator analyses results indicated that a student's educational level and the ITI measurement, specifically, the ITI measure used, the general or specific-subject based ITI assessment, the original or adapted version of the instrument, and the cultural background, moderated the link between the incremental and entity beliefs of intelligence and students' academic achievement. In this study, the predictive value of gender on the association of ITI and achievement was not confirmed, contrary to some evidence in the literature (Dweck, 1999; Pepi et al., 2006).

### Students' educational levels

Globally, the results indicated that students with more dynamic beliefs of intelligence are more likely to earn higher grades in the different academic cycles from middle school to college. In particular, the moderator effect of educational level is more evident in the middle school, where the association of incremental theories and achievement attained a moderate magnitude. In the present meta-analytic review, there was a significant positive association of incremental perspectives and verbal and quantitative subjects' grades and global achievement (*r* = 0.19 to 0.21, *p* < 0.001).

In fact, during early stages of adolescence, there is an intensive intellectual development, including meta-cognition and independent thought (Stevenson, 2002; Kellough and Kellough, 2008). Moreover, in these stages, students become very motivated to learn topics of their interest, and they start defining beliefs about themselves through introspection (Brighton, 2007). As learners, they will build upon their individual experiences and prior knowledge to conceive their world (Piaget, 1960). Therefore, these results support the fact that in the middle school learners begin to define academic performance objectives and individual strategies to accomplish them. Develop efforts to reinforce students' incremental perspective of intelligence, through experiential strategies, can constitute a significant step to foster academic achievement.

Additionally, the interesting result found in this study with respect to the negative verbal grade association (*r* = −0.21, *p* = 0.004) with the incremental perspective (which corresponds theoretically to a more entity-based perspective of intelligence) might indicate children's tendency to perceive the verbal domain, which is related to their mother tongue, to be a less challenging subject in which less effort is needed and where working for outcomes could be an adaptive strategy for students. In line with these results, in the high school context, a significant positive association between entity beliefs and the students' verbal achievement was also found (although low *r* = 0.08, *p* < 0.016).

### ITI measurement

The use of ITI assessment, both by incremental and entity theories scales, based in specific academic subjects or in their ability to perform well in a specific subject were, as expected, more related to general academic achievement (*r* = 0.13 to −0.27, *p* < 0.001) and to quantitative academic domains (*r* = 0.16, *p* < 0.001) than general ITI scales' assessment. As argued previously, ITI can vary across academic subjects (Dweck, 1999), since students can attribute different value and expectancy to their performance in the academic domains. Thus, ITI measurement based in the specific ability to perform in a determined academic subject provides students with the possibility to more precisely report their beliefs, which will likely influence the greater magnitude association with academic performance (Bandura, 1997, 2006).

In particular, concerning the instrument used to assess ITI and considering the limited number of studies with different measures, the moderator analyses indicated that the association of ITI and academic achievement is moderated overall by the Dweck's scales (version 1999, *r* = 0.13 version 2006, *r* = 0.35, *p* < 0.001), followed at a lesser magnitude by Faria's (2006) instrument, developed based on Dweck's work. Although the limitation of these analyses is assumed, the results highlighted the tendency to the recent versions of ITI scales to be more associated with achievement. These results may be because the measures' adapted versions were subjected to development and refinement, and by consequence of the process, instruments' validity in general could have increased, and the predictive validity of ITI in particular.

Moreover, the results related to the moderator effect of the use of the original ITI instrument and the use of a measure that has been translated and adapted to other context, revealed that when using the original instrument is more likely to achieve a greater association between both incremental and entity beliefs and academic achievement (*r* = |0.10 to 0.22|, *p* < 0.01). As anticipated, the demanding procedure of translation and adaptation of an instrument to other language and culture requires, in some instances, the additional processes of refinement and development. In this sense, the original ITI measure developed within a certain population is more likely to capture the effect for which it was initially developed.

### Cultural background

The moderator effect that students' cultural background could have on the association of ITI and their achievement was explored. The results indicated that considering the number of countries included in this meta-analysis, both Asia and Oceania (Australia) reported a significant association between incremental beliefs and students' achievement (*r* = 0.12 to 0.21, *p* < 0.001), while the entity perspective was not associated with achievement. These results obtained in Eastern continents might reflect the cultural differences at the educational level, for instance, as more collectivist societies might focus less in individual results and encourage students to value the learning process over academic achievement. On the other hand, in Europe, entity or fixed beliefs of intelligence were modestly associated with achievement, yet positively, in particular, in specific academic subjects (verbal and quantitative; *r* = 0.07 to 0.08, *p* < 0.02). As mentioned, in Europe there is a tendency toward a more academically and professionally competitive society, which could influence the students' perspectives of intelligence, leading them to prioritize individual outcomes and to value positive assessment over knowledge (Elliott and Dweck, 1988; Robins and Pals, 2002). In North America, in general, only the entity beliefs were negatively correlated with students' achievement (*r* = −0.22, *p* < 0.001), indicating that students with fixed conceptions about intelligence are more likely to have lower grades. In this continent, the results indicated that having an entity perspective of intelligence was more prejudicial for students' achievement than an incremental one was advantageous.

### Limitations and implications

This meta-analysis is not free from limitations. First, only the verbal, quantitative and general assessment grades (both objective or self-reported) were selected as indicators of students' academic achievement. Other academic subjects related to science or arts, which also implies significant effort from student's were not found in a significant number to be included in this study. Second, a limited number of studies separately explored the effect of students' entity theories or beliefs, which could have confirmed the impact that these specific beliefs have on academic achievement and performance. Third, the several ITI measures used in the studies reviewed were mostly based on Dweck's TIS scale, which might have led some of the results to overlap. Fourth, all the reports reviewed explored only the direct effect of ITI and academic achievement, and thus, further research should include indirect effects of the studied variables. Moreover, in the present meta-analytic review the nature of study's design (e.g., experimental, cross-sectional, longitudinal) was not explored, which could have had influence on the given effect size's. Fifth, this meta-analysis explored the potential moderator effects that gender, academic grade, ITI measurement and culture have in the link between ITI and academic achievement. Nonetheless, other variables, such as students' socioeconomic status, previous records of failing or type of school students attend to could have been explored as possible moderators of this association. Moreover, in future studies, cognitive or aptitude tests, and subjective indicators of academic outcomes, as alternative indicators of students' performance could also deepen and narrow the impact of ITI on students' outcomes.

Additionally, the results stressed the fact that students' educational level and cultural background can influence the ITI association with academic achievement. The fact that today schools need to address a diverse group of students with different educational needs, have an increasing pressure to achieve better academic results (mostly assessed by standardized quantitative testing), and ultimately to prepare students to succeed not only academically but also to have meaningful lives, has led to changes in the educational systems all over the world. Thus, to include a broader perspective of the studies' educational system type in further research would possibly shed light on the results found in this study, related both to the differences in the students' educational levels demands and cultural educational contexts.

Furthermore, the results suggest important implications for educational settings. As discussed in previous studies (Jaeggi et al., 2014; Alesi et al., 2016), the implementation and development of target-interventions or programs for improving academic achievement should consider the development of both domain-specific academic contents and the students' motivational intraindividual patterns of response, in order to achieve and sustain potential enhancements. Additionally, these interventions should be conducted at early stages of academic goals setting in children, where the link between incremental beliefs and achievement is particularly stronger.

## Conclusions

In this article, incremental and entity theories of intelligence drew together two relatively independent research results; however, both theories influenced aspects of students' academic achievement. Incremental theories were generally positively associated with different student's academic outcomes, whereas the entity theories obtained the highest impact on students' achievement, either positively or negatively. Additionally, ITI were more strongly associated with academic achievement when ITI's measurement considered specific academic subjects, providing evidence for the more predictive validity of implicit theories when capturing the specific beliefs of performance in different academic domains.

This study highlighted the fact that the different types of ITI measurement can produce different associations with students' achievement. Additionally, the cross-cultural ITI effects could reflect the underlying differences in conceptualization of the construct across countries. This meta-analysis updates the current evidence supporting the direct link of ITI and students' achievement and acknowledges specific effects that ITI could have on students' academic outcomes. However, further research is still needed to clarify measurement and conceptual issues underlying this relationship. Nonetheless, the present paper supports the importance of students' motivational patterns in the definition and operationalization of their academic path.

## Author contributions

Both authors contributed directly and significantly to this study. AC and LF developed the idea for the study. AC performed the codification of the studies, data analyses, and contributed to paper writing. LF contributed to the revision of the manuscript. Both authors read and approved the manuscript.

### Conflict of interest statement

The authors declare that the research was conducted in the absence of any commercial or financial relationships that could be construed as a potential conflict of interest.
